# ADAMO INDOOR MOBILITY, PHYSICAL FRAILTY, AND AUTONOMY IN OLDER ADULTS: A MEDIATION MODEL

**DOI:** 10.1093/geroni/igz038.2516

**Published:** 2019-11-08

**Authors:** Alberto Rainoldi, Lorenzo M Donini, Paolo R Brustio, Anna Mulasso, Eleonora Poggiogalle, Gianluca Zia, Luca C Feletti, Susanna Del Signore

**Affiliations:** 1 University of Turin, Italy - Neuro Muscular Function Research group, School of Exercise and Sport Sciences, Department of Medical Sciences, Torino, Italy; 2 Sapienza University, Rome, Italy; 3 University of Turin - NeuroMuscularFunction research group School of Exercise and Sport Sciences Department of Medical Sciences, Torino, Italy; 4 Bluecompanion LTD, London, England, United States; 5 Caretek srl, Torino, Italy; 6 Bluecompanion LTD, United Kingdom, France

## Abstract

Physical frailty represents a clinical condition among older adults leading to adverse health outcomes, such as autonomy loss. To evaluate physical frailty in older adults, adopting information and communication technologies (ICT) may be useful. ADAMO (Caretek S.r.l.) is a care-watch accelerometer that allows to measure mobility in a non-intrusive way (Magistro et al., 2018) providing wider information on individual general health (Mulasso et al., in press). The aim of this study was to evaluate the relationship between indoor mobility, physical frailty and autonomy in a sample of Italian older adults. Methods: Thirty-two volunteers (age 65–84 years; women 56.2%) participated in the study. All wore ADAMO care-watch continuously over a 7-day period. The number of steps indoor was the main endpoint. Fragmented daily mobility was estimated. Physical frailty and autonomy were measured using the Tilburg Frailty Indicator (physical components) and the Groningen Activity Restriction Scale, respectively. Results: Significant inverse correlations were observed between number of steps and autonomy, and number of steps and physical frailty. Conversely, a significant direct correlation was observed between physical frailty and autonomy. Additionally, mediation analysis demonstrated full mediation effect of physical frailty between the number of steps and autonomy. Our results imply that high indoor mobility per se can reduce physical frailty and consequently helps to maintain autonomy. Conclusions: Indoor mobility captured by ADAMO accelerometer may be an important indicator of physical frailty and autonomy. ADAMO may be used as a non-intrusive telemonitoring solution to capture relevant information on individual general health in aged people.

